# Sequential NIHSS–NLR monitoring after endovascular thrombectomy: malignant cerebral oedema and inflammatory futility in a two-phase derivation study

**DOI:** 10.3389/fneur.2026.1898491

**Published:** 2026-09-03

**Authors:** Hesham A. Alyamani, Anter M. A. Nasser, Qin Huang, Nabeel M. Abdo, Yinghuan Pan, Nazakat Nurmamat, Bi Deng, Jian Xia

**Affiliations:** 1 Department of Neurology, Xiangya Hospital, Central South University, Changsha, China; 2 Department of Neurology, Thamar University, Dhamar, Yemen; 3 Department of Cardiology, Xiangya Hospital, Central South University, Changsha, China; 4 Clinical Research Center for Cerebrovascular Disease of Hunan Province, Changsha, China

**Keywords:** decision curve analysis, derivation study, endovascular thrombectomy, futile recanalisation, malignant cerebral oedema, net reclassification improvement, neutrophil-to-lymphocyte ratio, NIHSS trajectory

## Abstract

**Introduction:**

Successful reperfusion after endovascular thrombectomy (EVT) does not guarantee recovery. Malignant cerebral oedema (MCE) complicates approximately one in eight patients, and static pre-procedural models do not capture evolving post-reperfusion risk. We evaluated a two-phase sequential clinical-inflammatory monitoring framework.

**Methods:**

We conducted a retrospective consecutive-patient cohort study at Xiangya Hospital (2021-2025) including 306 successfully reperfused EVT patients. Phase 1 combined the 24-h NIHSS trajectory with neutrophil-to-lymphocyte ratio (NLR) direction for clinically ascertained MCE risk. Ridge-penalised logistic models were internally validated using Harrell bootstrap correction. Exploratory Phase 2 combined 72-h NLR with CT-perfusion anatomy for futile recanalisation (90-day mRS ≥3).

**Results:**

MCE occurred in 39 patients (12.7%), all co-occurring with early neurological deterioration. NIHSS worsening was the dominant predictor (aOR 6.95, 95% CI 3.63-14.46; *E*-value 13.4; corrected AUC 0.791). NLR direction provided minimal incremental discrimination (DeltaAUC +0.008, 95% CI -0.007 to +0.020) but separated complication profiles: concurrent NIHSS worsening and NLR rise identified an oedema-enriched subgroup (MCE 26.9%; NNM 3.7), whereas non-rising NLR among worsening patients identified a haemorrhage-enriched subgroup (sICH 37.7%). At 72 h, favourable anatomy with elevated NLR defined a candidate inflammatory futility phenotype with lower functional independence (57.3% vs 84.2%; corrected AUC 0.631, upper bound by construction).

**Discussion:**

Because MCE ascertainment was clinically triggered and adjudication was unblinded to NIHSS trajectory, Phase 1 identifies a clinical-inflammatory risk pattern rather than an independent radiological predictor. NLR direction functions primarily as a complication-profile stratifier. Prospective multicentre validation with blinded radiological adjudication is required before clinical or trial-design application.

## Introduction

Successful reperfusion after endovascular thrombectomy (EVT) does not guarantee biological or clinical recovery. A subset of patients deteriorates despite technically successful recanalisation, developing malignant cerebral oedema (MCE), haemorrhagic complications, or later futile recanalisation. MCE—defined as space-occupying hemispheric infarction with midline shift ≥5 mm, infarction exceeding 50% of the MCA territory, clinical herniation syndrome, the requirement for decompressive hemicraniectomy, or oedema-attributable death (1)—is the most immediately life-threatening of these complications. MCE complicates approximately one in eight patients undergoing thrombectomy, carries a case-fatality rate exceeding 50%, and may not be radiologically apparent until the scheduled 60–84-h ascertainment window, long after the monitoring decisions that determine clinical outcome have been made (2–6). Futile recanalisation, defined as technical success followed by 90-day functional dependence, occurs in approximately one third of EVT cases and is not fully explained by baseline CT-perfusion anatomy alone (7, 8).

Current risk-stratification approaches remain largely static. Most available MCE prediction models rely on pre-procedural clinical, imaging, or technical variables and achieve discriminative performance with AUCs of 0.60–0.70 across systematic reviews (9–11). None provides actionable guidance at 24 h—approximately 36–60 h before radiological confirmation—when monitoring decisions determine outcome. Neurological trajectory at 24 h captures evolving clinical deterioration, whereas the neutrophil-to-lymphocyte ratio (NLR) provides a readily available marker of systemic inflammatory response that may change before CT consequences become apparent (12–16). The direction of NLR change carries more clinical information than its absolute value at any single measurement. Among neurologically worsening patients, a rising NLR from a low admission baseline reflects post-reperfusion neutrophil mobilisation and oedema-enriched deterioration; a non-rising NLR from an already-elevated baseline signals a haemorrhagic injury pattern driven by pre-existing systemic inflammatory activation. These patterns are grounded in post-reperfusion neutrophil biology: a rising NLR from a low baseline is consistent with the acute neutrophil mobilisation described by Gelderblom et al. (12) and Wang et al. (13), which drives blood–brain barrier disruption and vasogenic oedema amplification, whereas a non-rising NLR from an already-elevated baseline reflects pre-existing systemic inflammatory activation linked to haemorrhagic vulnerability by Pikija et al. (17) and Zheng et al. (18). The directional distinction within the neurologically worsening subgroup has not been previously validated and constitutes a novel hypothesis requiring prospective confirmation. This distinction is further amplified in intracranial atherosclerotic disease (ICAD)-management–predominant EVT populations, where neutrophil count mediates the relationship between atherosclerotic plaque burden and procedural outcomes, and where rescue angioplasty, intracranial stenting, and tirofiban exposure superimpose local arterial inflammatory stimuli onto the systemic ischaemia–reperfusion response (19–21).

We therefore evaluated a two-phase, temporally ordered clinical–inflammatory monitoring framework in a consecutive single-centre derivation cohort of patients with successful recanalisation after EVT. The primary Phase 1 objective was to determine whether a 24-h NIHSS trajectory combined with NLR direction stratifies clinically ascertained MCE risk before the 60–84-h ascertainment window. The secondary exploratory Phase 2 objective was to examine whether 72-h NLR combined with CT-perfusion anatomy identifies candidate phenotypes of futile recanalisation. The conceptual framework is illustrated in Supplementary Figure S2.

## Methods

### Study design and participants

**Figure 1:**
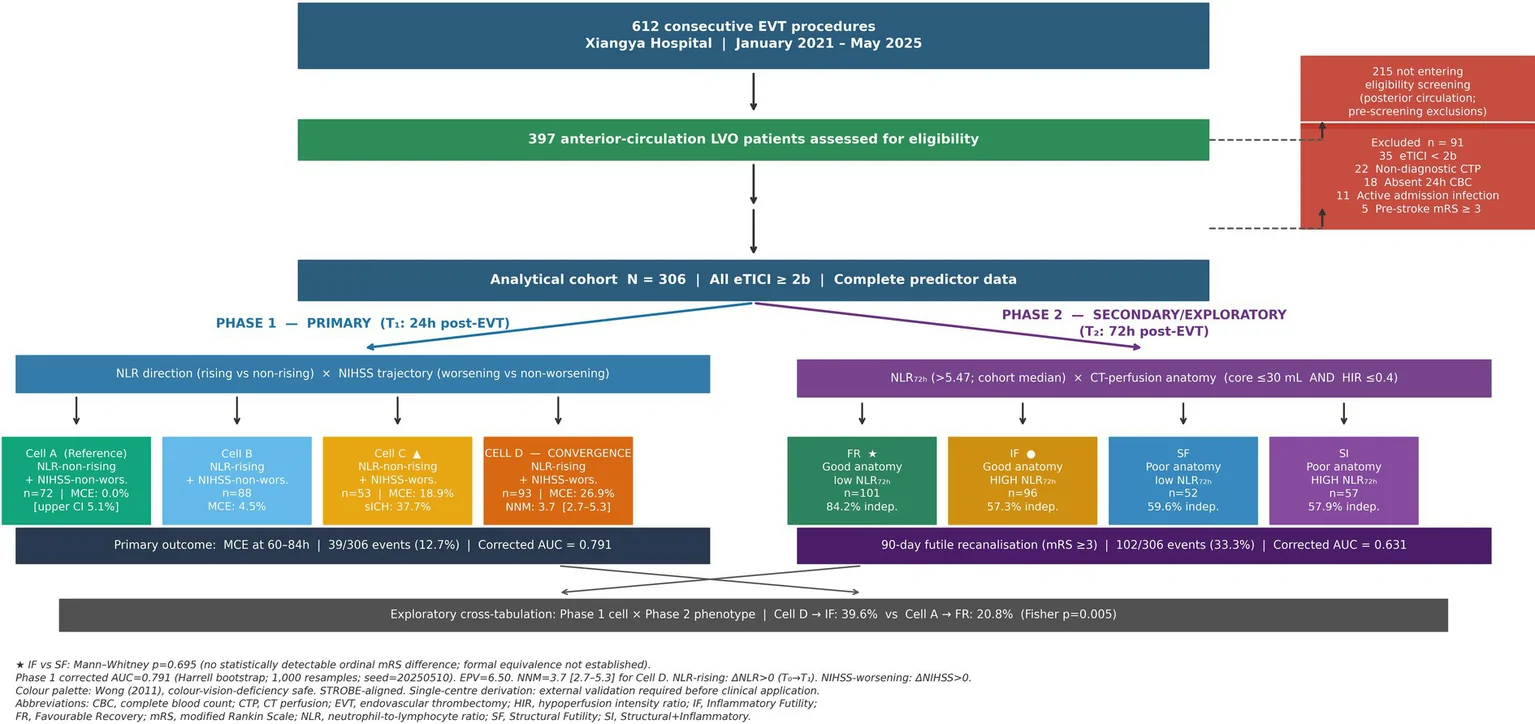
STROBE-aligned patient flow and two-phase classification architecture. Of 612 consecutive EVT procedures at Xiangya Hospital (2021–2025), 397 anterior-circulation LVO patients were assessed for eligibility; 91 were excluded (35 eTICI <2b; 22 non-diagnostic CTP; 18 absent 24-h CBC; 11 active infection at admission; 5 pre-stroke mRS ≥ 3), leaving 306 patients in the analytical cohort. Posterior-circulation cases, incomplete records, and repeat procedures were among the 215 not assessed. The diagram illustrates the Phase 1 24-h NIHSS–NLR four-cell classification and the Phase 2 72-h anatomy-stratified inflammatory phenotyping structure. CBC, complete blood count; CTP, CT perfusion; EVT, endovascular thrombectomy; LVO, large-vessel occlusion; MCE, malignant cerebral oedema; mRS, modified Rankin Scale; NLR, neutrophil-to-lymphocyte ratio; NIHSS, National Institutes of Health Stroke Scale.

We conducted a retrospective consecutive-patient cohort study at Xiangya Hospital, Central South University, from January 2021 to May 2025 (ethics approval No. 202304068; written informed consent waived; the 2013 revision of the Declaration of Helsinki was followed; STROBE-aligned (22)). Among 612 consecutive EVT procedures, 397 anterior-circulation large-vessel occlusion (LVO) patients who completed the procedure were assessed for eligibility; 215 were not assessed because of posterior-circulation occlusion, incomplete records, or repeat procedures at the same admission. This cohort comprised exclusively anterior-circulation LVO patients; posterior-circulation cases were identified at screening and excluded from eligibility assessment. The NIHSS-worsening definition is therefore applied within a population for which the NIHSS has established clinical validity and sensitivity for hemispheric stroke. We excluded 91 patients: 35 with eTICI <2b, 22 with non-diagnostic CT perfusion (CTP), 18 without a 24-h complete blood count (CBC), 11 with active infection at admission, and 5 with pre-stroke modified Rankin Scale (mRS) score ≥3. The analytical cohort comprised 306 patients, all with complete Phase 1 predictor data (Figure 1). Atrial fibrillation (AF) status was confirmed completely after source-record review.

The cohort was ICAD-management–predominant, operationally defined by rescue angioplasty, intracranial stenting, or intra-procedural tirofiban in 170 of 306 patients (55.6%); ICAD was not separately adjudicated as a standalone stroke mechanism. Of the 91 excluded patients, 40 had missing predictor data (absent 24-h CBC: *n* = 18; non-diagnostic CTP: *n* = 22); individual-level data for these patients were not retained, and baseline comparisons with the analytical cohort could not be performed. With 39 MCE events and 6 candidate predictors, the events-per-variable ratio (EPV) was 6.50; ridge penalisation and bootstrap optimism correction were applied accordingly (23).

### Phase 1: 24-h clinical–inflammatory classification

The admission NLR (T₀) was derived from the pre-procedure complete blood count; the 24-h NLR (T₁) was from the routine blood count obtained 22–26 h after the end of the EVT procedure. NLR-rising was defined as ΔNLR > 0; NLR-non-rising as ΔNLR ≤0. NIHSS-worsening was defined as ΔNIHSS > 0; NIHSS-non-worsening as ΔNIHSS ≤0. The four Phase 1 cells were assigned at T₁, approximately 36–60 h before the MCE ascertainment window.

The primary outcome was clinically ascertained MCE at 60–84 h per HAMLET, DECIMAL, and DESTINY criteria (1): midline shift ≥5 mm, infarction >50% of MCA territory, clinical herniation syndrome, requirement for decompressive hemicraniectomy, or oedema-attributable death. Two independent neurologists adjudicated events with consensus review; adjudicators were not blinded to NIHSS trajectory. All 39 MCE events co-occurred with early neurological deterioration (END), defined as NIHSS increase ≥4 by 72 h (24), precluding a purely radiological sensitivity analysis; this limitation is addressed in the Discussion. Safety outcomes were symptomatic intracranial haemorrhage (sICH) per ECASS-II criteria and END (25).

### Phase 2: 72-h anatomy-stratified inflammatory phenotyping (secondary/exploratory)

Favourable anatomy was defined from the pre-treatment CT-perfusion scan as core volume ≤30 mL AND hypoperfusion intensity ratio (HIR) ≤ 0.4, informed by the hypoperfusion-intensity literature (26–28), and was not intended to replicate DAWN or DEFUSE-3 eligibility criteria (4, 29). The 72-h NLR (NLR₇₂ₕ) was obtained from the blood count drawn 66–80 h after the procedure; in the absence of an established post-EVT threshold, it was dichotomised at the cohort median (5.47) to maximise phenotype balance (see Limitations). Four phenotypes were defined by crossing anatomy status (favourable or unfavourable) with NLR₇₂ₕ status (low or elevated): favourable recovery, inflammatory futility, structural futility, and structural-plus-inflammatory. The Phase 2 endpoint was futile recanalisation (90-day mRS ≥ 3).

### Statistical analysis

Phase 1 used nine predefined ridge-penalised logistic regression models (M0–M8; L2 *λ* = 1.0 fixed to stabilise estimates under low EPV; Python 3.12, scikit-learn 1.4.2, scipy 1.13.0, statsmodels 0.14.1; seed 20250510). The primary model M6 included core volume, baseline NIHSS, age, onset-to-groin time (standardised per SD), NLR-rising, and NIHSS-worsening. Internal validation used Harrell bootstrap optimism correction (1,000 resamples); bootstrap 95% CIs used 2,000 iterations; paired AUC differences used 5,000 iterations (23). Single-predictor AUCs are apparent values; multivariable AUCs are Harrell-corrected; benchmark comparisons are descriptive.

The following analyses were pre-specified in the statistical analysis plan: the nine ridge-penalised model tiers (M0–M8), categorical net reclassification improvement (NRI) at 10, 20, and 30% thresholds, the AF × NIHSS-worsening interaction test, and the Phase 2 sensitivity analysis excluding patients with MCE or sICH. The following were post-hoc and exploratory: cross-phase linkage analysis (Supplementary Table S3), benchmark comparisons with single-predictor AUCs (Supplementary Table S4), alternative NLR operationalisations (Supplementary Table S5), and Cell D core-volume stratification (Supplementary Table S6). The complete Python 3.12 analysis code with seed and standardisation parameters is available upon request to permit full replication of all reported analyses.

Calibration was assessed using Harrell-corrected slope, intercept, Brier score, and Hosmer–Lemeshow goodness-of-fit statistic (10 groups) (23). Clinical utility was evaluated by decision curve analysis across threshold probabilities of 5–50% (30). Reclassification was assessed by categorical NRI at 10, 20, and 30% thresholds—pre-specified to reflect clinically relevant monitoring intensity levels at the observed 12.7% MCE rate—and by continuous NRI (2,000 bootstrap CIs). Unmeasured confounding was quantified using *E*-values (31). The pre-specified AF × NIHSS-worsening interaction was evaluated by a likelihood ratio test. The full M6 prediction equation with standardisation parameters is provided in Supplementary Table S2 (TRIPOD items 15a/15b) (32). The prospective validation sample size was estimated for 80% power to confirm AUC ≥ 0.70 (*α* = 0.05) (33).

Phase 2 logistic regression estimated phenotype-outcome associations with AF as a covariate. The anatomy × NLR72h interaction was evaluated using both a Woolf test on the 2 × 2 phenotype table and a likelihood ratio test with NLR72h as a continuous predictor. Harrell-corrected AUC used the same 1,000-resample bootstrap procedure as Phase 1. A prespecified sensitivity analysis excluded patients with MCE or sICH (*n* = 228). All 306 patients were included as AF status was complete after source-record review.

## Results

### Cohort characteristics

**Figure 2:**
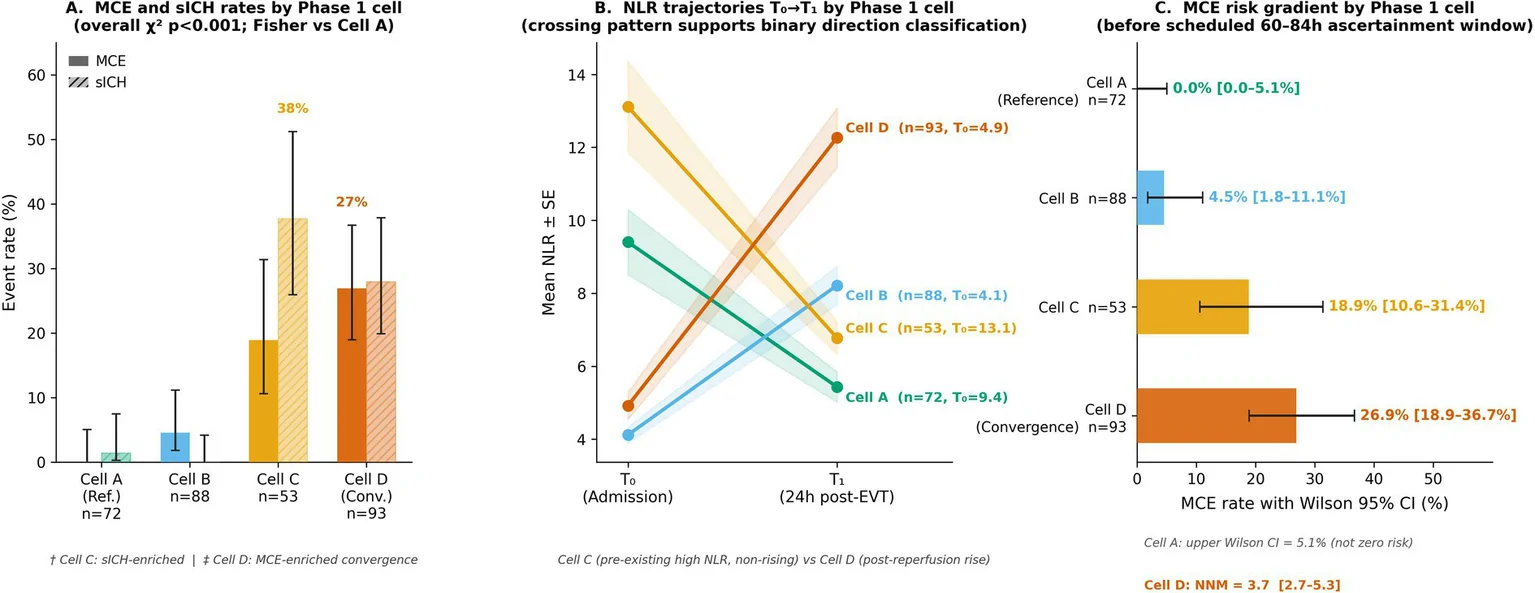
Phase 1 24-h monitoring: MCE risk gradient, NLR trajectories, and complication-profile stratification (*N* = 306). **(A)** MCE and sICH proportions across the four NIHSS–NLR cells; Fisher’s exact test vs. Cell A; overall *χ*^2^ *p* < 0.001. **(B)** NLR trajectories from admission to 24 h; the diverging trajectories of Cell C (pre-existing high NLR, non-rising) and Cell D (low baseline, post-reperfusion rise) illustrate why NLR direction encodes information beyond the isolated NLR value. **(C)** MCE risk gradient by cell with Wilson 95% CIs; Cell A upper bound 5.1% annotated; NNM for Cell D = 3.7 [2.7–5.3]. MCE, malignant cerebral oedema; NLR, neutrophil-to-lymphocyte ratio; NIHSS, National Institutes of Health Stroke Scale; sICH, symptomatic intracranial haemorrhage.

Among 306 patients in the analytical cohort, the median age was 65 years [IQR 58–72], 212 (69.3%) were male, and the median baseline NIHSS score was 12 [IQR 8–16]. At 24 h, 181 patients (59.2%) had a rising NLR, and 146 (47.7%) had NIHSS worsening. Baseline NLR differed across the four Phase 1 cells: the lowest median was in Cell D (3.98), reflecting a predominantly post-procedural inflammatory state, and the highest in Cell C (11.86), reflecting pre-existing systemic inflammation. By 24 h, Cell D showed a post-reperfusion NLR rise, whereas Cell C showed a stable or falling trajectory. Baseline characteristics are summarised in Table 1, and NLR trajectories are shown in Figure 2B.

**Table 1:** Baseline characteristics by Phase 1 (24-h) cell assignment (*N* = 306).

Variable	Reference (NLR-non-rising + NIHSS-non-worsening) *n* = 72	NLR-rising + NIHSS-non-worsening *n* = 88	NLR-non-rising + NIHSS-worsening *n* = 53	Convergence (NLR-rising + NIHSS-worsening) *n* = 93	Total *N* = 306	*p*-value
Demographics						
Age, years	62.5 [56.8–71.0]	64.0 [57.0–70.0]	66.0 [58.0–74.0]	65.0 [59.0–73.0]	65.0 [58.0–72.0]	0.418
Male sex	54 (75.0%)	61 (69.3%)	33 (62.3%)	64 (68.8%)	212 (69.3%)	0.505
BMI, kg/m^2^	25.4 [23.8–27.1]	25.6 [23.6–27.4]	24.5 [23.3–27.2]	26.2 [24.0–27.6]	25.4 [23.6–27.4]	0.185
Clinical						
Hypertension	53 (73.6%)	58 (65.9%)	33 (62.3%)	74 (79.6%)	218 (71.2%)	0.084
Diabetes mellitus	21 (29.2%)	26 (29.5%)	13 (24.5%)	33 (35.5%)	93 (30.4%)	0.556
Atrial fibrillation	13 (18.1%)	25 (28.4%)	16 (30.2%)	15 (16.1%)	69 (22.5%)	0.089
Smoker	38 (52.8%)	40 (45.5%)	22 (41.5%)	50 (53.8%)	150 (49.0%)	0.409
Prior stroke	9 (12.5%)	20 (22.7%)	6 (11.3%)	14 (15.1%)	49 (16.0%)	0.209
Baseline NIHSS	11.0 [7.0–14.0]	12.0 [7.0–16.0]	14.0 [11.0–19.0]	13.0 [8.0–17.0]	12.0 [8.0–16.0]	0.003
Baseline pulse pressure, mmHg	57.0 [50.0–68.2]	55.0 [50.0–70.0]	58.0 [50.0–68.0]	58.0 [50.0–68.0]	57.0 [50.0–69.0]	0.884
Admission glucose (mmol/L)	6.8 [5.8–8.9]	6.1 [5.5–7.6]	7.0 [6.0–9.1]	6.6 [5.5–8.1]	6.6 [5.6–8.2]	0.032
Imaging						
Ischaemic core volume, mL	2.8 [0.0–24.8]	3.8 [0.0–16.7]	12.6 [0.9–40.8]	7.0 [0.0–35.0]	6.5 [0.0–30.2]	0.026
Hypoperfusion intensity ratio (HIR)	0.2 [0.0–0.4]	0.2 [0.0–0.4]	0.3 [0.0–0.4]	0.1 [0.0–0.4]	0.2 [0.0–0.4]	0.493
Mismatch volume, mL	114.4 [66.8–156.8]	90.6 [60.3–151.4]	94.7 [53.0–159.0]	76.9 [46.0–122.0]	91.6 [54.3–145.4]	0.058
Good anatomy (core ≤30 mL AND HIR ≤ 0.4)	49 (68.1%)	62 (70.5%)	27 (50.9%)	59 (63.4%)	197 (64.4%)	0.109
Laboratory						
NLR at admission (T₀)	6.7 [4.4–11.4]	3.9 [2.4–5.3]	11.9 [7.3–17.0]	4.0 [2.5–5.5]	5.0 [3.1–8.4]	<0.001
NLR at 24 h (T₁)	4.5 [3.1–6.4]	7.1 [4.5–10.6]	6.4 [4.5–8.4]	9.5 [6.7–16.6]	6.9 [4.5–10.8]	<0.001
NLR at 72 h (T₂)	3.9 [2.9–5.5]	5.2 [3.4–6.9]	7.6 [5.5–10.4]	6.9 [4.7–12.5]	5.5 [3.6–9.0]	<0.001
Procedural						
Received IVT	20 (27.8%)	17 (19.3%)	12 (22.6%)	26 (28.0%)	75 (24.5%)	0.495
Onset-to-groin time, min	405.0 [287.5–542.5]	375.0 [240.0–615.0]	480.0 [300.0–660.0]	420.0 [240.0–600.0]	420.0 [250.0–600.0]	0.378
Procedure time, min	120.0 [93.8–140.0]	120.0 [90.0–155.8]	130.0 [100.0–150.0]	135.0 [100.0–165.0]	125.0 [100.0–151.0]	0.109
Rescue angioplasty	34 (47.2%)	42 (47.7%)	19 (35.8%)	46 (49.5%)	141 (46.1%)	0.424
Intracranial stenting	27 (37.5%)	26 (29.5%)	12 (22.6%)	38 (40.9%)	103 (33.7%)	0.103
Tirofiban	23 (31.9%)	24 (27.3%)	18 (34.0%)	20 (21.5%)	85 (27.8%)	0.325
Outcomes						
MCE at 60–84 h	0 (0.0%)	4 (4.5%)	10 (18.9%)	25 (26.9%)	39 (12.7%)	<0.001
sICH within 72 h	1 (1.4%)	0 (0.0%)	20 (37.7%)	26 (28.0%)	47 (15.4%)	<0.001
END within 72 h	1 (1.4%)	6 (6.8%)	45 (84.9%)	79 (84.9%)	131 (42.8%)	<0.001
Futile recanalisation (mRS ≥ 3 at 90 d)	15 (20.8%)	15 (17.0%)	28 (52.8%)	44 (47.3%)	102 (33.3%)	<0.001
Functional independence (mRS 0–2 at 90 d)	57 (79.2%)	73 (83.0%)	25 (47.2%)	49 (52.7%)	204 (66.7%)	<0.001

Values are median [IQR] for continuous variables and *n* (%) for categorical variables unless stated.

END, early neurological deterioration; HIR, hypoperfusion intensity ratio; IVT, intravenous thrombolysis; MCE, malignant cerebral oedema; mRS, modified Rankin Scale; NLR, neutrophil-to-lymphocyte ratio; sICH, symptomatic intracranial haemorrhage. *p*-values: Kruskal–Wallis for continuous variables; chi-squared for categorical variables.

### Phase 1 primary analysis

Clinically ascertained MCE occurred in 39 patients (12.7%), sICH in 47 (15.4%), and END in 131 (42.8%). All 39 MCE events co-occurred with END; no MCE event arose in patients without END, precluding a radiological-only sensitivity analysis.

The four-cell 24-h clinical–inflammatory classification showed a graded MCE-risk gradient. The convergence cell (Cell D; NLR-rising + NIHSS-worsening; *n* = 93) had an MCE proportion of 26.9% [Wilson 95% CI 18.9–36.7%]; the bootstrap OR vs. the reference cell was 7.83 [2.38–25.73], *p* < 0.001; NNM 3.7 [2.7–5.3]. Cell B (NLR-rising + NIHSS-non-worsening; *n* = 88) had MCE 4.5%. The reference cell (Cell A; NLR-non-rising + NIHSS-non-worsening; *n* = 72) had no MCE events (Wilson upper bound 5.1%). Among patients with NIHSS worsening, complication profiles diverged by NLR direction: Cell D showed near-equal MCE and sICH rates (26.9% vs. 28.0%), whereas Cell C (NLR-non-rising + NIHSS-worsening; *n* = 53) showed a haemorrhage-enriched pattern [sICH 37.7% (25.9–51.2%) vs. MCE 18.9%]. The overall four-cell association was significant, *χ*^2^ *p* < 0.001 (Table 2; Figures 2A,C).

**Table 2:** Phase 1 (24-h) MCE prediction matrix—primary analysis (*N* = 306).

Phase 1 cell	*n* (% of cohort)	MCE *n*/rate	MCE Wilson 95% CI	sICH n/rate	sICH Wilson 95% CI	END *n*/rate	vs Cell A *p*	Research interpretation for prospective validation
Cell A: NLR-non-rising + NIHSS-non-worsening (Reference cell)	72 (23.5%)	0/0.0%	[0.0–5.1%]	1/1.4%	[0.2–7.5%]	1/1.4%	— (reference)	Candidate for the lowest-risk stratum in prospective studies; Zero events observed, but Wilson’s upper bound 5.1%—not zero risk; adequate monitoring still required
Cell B: NLR-rising + NIHSS-non-worsening	88 (28.8%)	4/4.5%	[1.8–11.1%]	0/0.0%	[0.0–4.2%]	6/6.8%	0.128	Low-risk stratum; NLR rise without NIHSS worsening insufficient for MCE risk escalation; candidate control-adjacent group
Cell C: NLR-non-rising + NIHSS-worsening	53 (17.3%)	10/18.9%	[10.6–31.4%]	20/37.7%	[25.9–51.2%]	45/84.9%	<0.001	Haemorrhagic pathway dominant (sICH 37.7%); candidate stratum for haemorrhagic transformation surveillance protocols in prospective studies
Cell D remains NLR-rising + NIHSS-worsening (convergence phenotype)	93 (30.4%)	25/26.9%	[18.9–36.7%]	26/28.0%	[19.9–37.8%]	79/84.9%	<0.001	Highest MCE-risk stratum (26.9%); candidate priority group for intensified oedema surveillance in prospective trials; ≈36–60 h before 60–84 h MCE ascertainment

END definition: NIHSS increment ≥4 from best post-procedural assessment at 72 h (not from T₀ admission). NIHSS trajectory classification uses a T₀ → T₁ (24 h) comparison, a different reference point. NIHSS-non-worsening Phase 1 cells can therefore contain END events occurring between T₁ (24 h) and T₂ (72 h). All pairwise Fisher’s exact p-values, two-sided vs. Cell A reference. MCE adjudication not blinded to NIHSS trajectory (disclosed limitation). END, early neurological deterioration; MCE, malignant cerebral oedema; NLR, neutrophil-to-lymphocyte ratio; sICH, symptomatic intracranial haemorrhage.

The research interpretation column replaces clinical-action language; this is a single-centre derivation study. All management decisions require prospective validation. MCE ascertained at 60–84 h post-EVT (HAMLET/DECIMAL/DESTINY criteria). Zero-event reference cell Wilson upper bound 5.1%: Wilson upper bound 5.1%: the zero-event reference cell does not establish zero risk.

**Figure 3:**
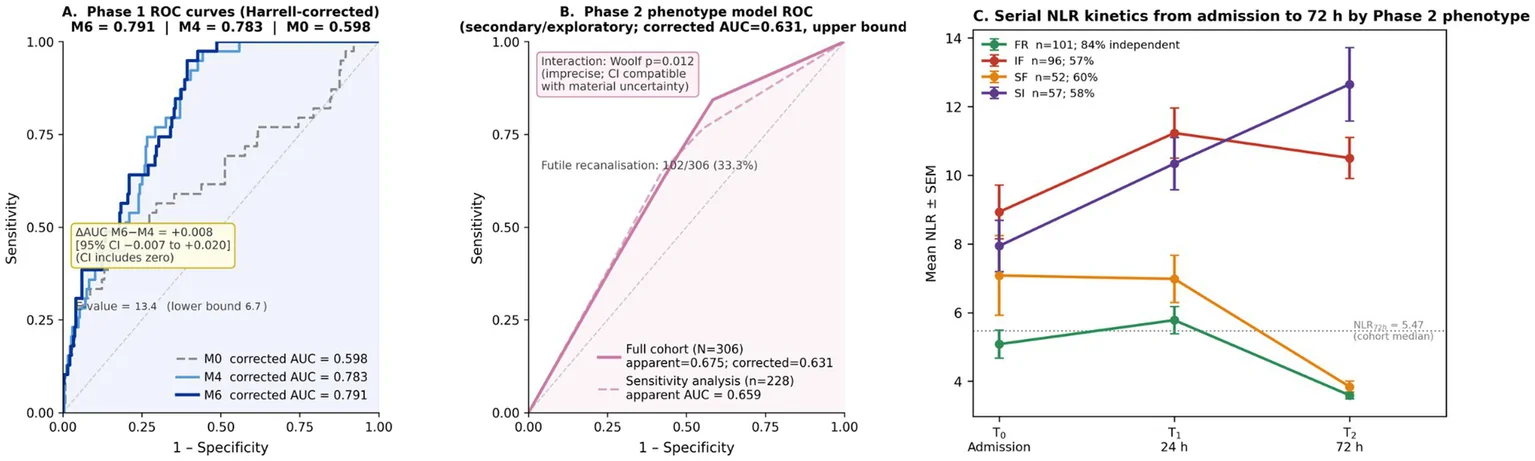
Model performance and serial NLR kinetics. **(A)** Phase 1 Harrell-corrected ROC curves for M0 (0.598), M4 (0.783), and M6 (0.791); ΔAUC M6 − M4 = +0.008 [−0.007 to +0.020] (95% CI crosses zero); *E*-value = 13.4; EPV = 6.50. Full prediction equation in Supplementary Table S2. **(B)** Phase 2 phenotype model ROC (secondary/exploratory); solid curve: full cohort (*N* = 306, apparent AUC 0.675, corrected AUC 0.631, upper bound); dashed curve: Sensitivity analysis excluding MCE/sICH patients (*n* = 228, apparent AUC 0.659). **(C)** Serial NLR kinetics from admission to 72 h by Phase 2 phenotype; dotted line marks the dataset-derived NLR₇₂ₕ threshold (5.47; cohort median). AUC, area under the curve; NLR, neutrophil-to-lymphocyte ratio; ROC, receiver operating characteristic.

In the primary ridge-penalised model, NIHSS worsening was the dominant predictor of clinically ascertained MCE [aOR 6.95 (3.63–14.46); *p* < 0.001; *E*-value 13.4, lower confidence bound 6.7]. NLR-rising showed a directional but non-significant association [aOR 1.77 (0.93–3.78); *p* = 0.105]. Core volume was independently associated with MCE [aOR 1.55 (1.15–2.19); *p* = 0.011]. Before bootstrap optimism correction, the apparent AUC was 0.638 for M0, 0.806 for M4, and 0.817 for M6; after Harrell correction (1,000 resamples), the corrected AUC was 0.598 for M0, 0.783 for M4, and 0.791 for M6, corresponding to a bootstrap optimism of 0.026 for the primary model. Incremental discrimination of M6 over M4 was minimal [ΔAUC +0.008 (−0.007 to +0.020)], whereas improvement over M0 was substantial (ΔAUC +0.193). Calibration of the apparent model was acceptable: apparent calibration slope 0.978, apparent calibration intercept −0.006, apparent Brier score 0.093 (Harrell-corrected Brier 0.0995), apparent Hosmer–Lemeshow *p* = 0.731 (Supplementary Table S2; Figure 3A). The Hosmer–Lemeshow statistic applied to Harrell-corrected predictions was *p* = 0.127, reflecting the distributional shift introduced by optimism correction in the predicted risk estimates.

Decision curve analysis showed higher apparent net benefit for M6 over both treat-all and treat-none strategies across threshold probabilities from 5 to 30%; net benefit differences vs. treat-all were +0.047 at 10%, +0.118 at 20%, and +0.269 at 30%. These estimates were derived from apparent model predictions and are not optimism-corrected.

### Supplementary phase 1 analyses

The following supplementary analyses are descriptive and hypothesis-generating; they are provided for transparency and to guide future prospective study design, not as primary findings.

In *post hoc* benchmark comparisons, HIR alone achieved apparent AUC 0.608, core volume alone 0.659, and NLR₂₄h alone 0.688 (34); M6 corrected AUC was 0.791. Because single-predictor AUCs were not separately corrected for optimism, benchmark comparisons are descriptive (Supplementary Table S4; Supplementary Figure S1). Categorical NRI of M6 vs. HIR alone was 0.658 [95% CI 0.382–0.899], with 56.4% of MCE patients correctly reclassified upward; NRI of M6 vs. NIHSS-worsening alone was 0.173 [0.007–0.343], which should be interpreted cautiously given the lower confidence bound approaches zero at EPV = 6.50 with only 39 events (Supplementary Table S7).

Alternative NLR operationalisations yielded nearly identical discrimination: continuous ΔNLR yielded AUC 0.796 and NLR ratio yielded 0.793, compared with 0.791 for binary NLR direction. Absolute NLR₂₄h yielded AUC 0.805 within M6 but was not pre-specified because it cannot reproduce the four-cell classification or the Cell C vs. Cell D complication-profile stratification (Supplementary Table S5).

Within Cell D, exploratory stratification by core volume ≥10 mL identified a higher-risk subgroup: MCE 39.0% [25.7–54.3%] vs. 17.3% [9.4–29.7%] among those with core volume <10 mL (Fisher *p* = 0.033; Bonferroni-adjusted *p* ≈ 0.13; NNM 2.6 [1.8–3.9]; hypothesis-generating) (Supplementary Table S6).

AF was present in 69 patients (22.5%). MCE rates were 10.1% in AF patients (7/69) and 13.5% in non-AF patients (32/237; Fisher *p* = 0.543). Cell D assignment occurred in 15/69 AF patients (21.7%) and 78/237 non-AF patients (32.9%). The NIHSS-worsening × AF interaction model was numerically unstable owing to sparse interaction cells; AF-related effect modification could not be reliably estimated.

### Phase 2 secondary exploratory analysis

The following results are entirely secondary and exploratory; they are presented to generate hypotheses for future prospective testing and should not be interpreted as carrying the same evidential weight as the primary Phase 1 findings.

**Figure 4:**
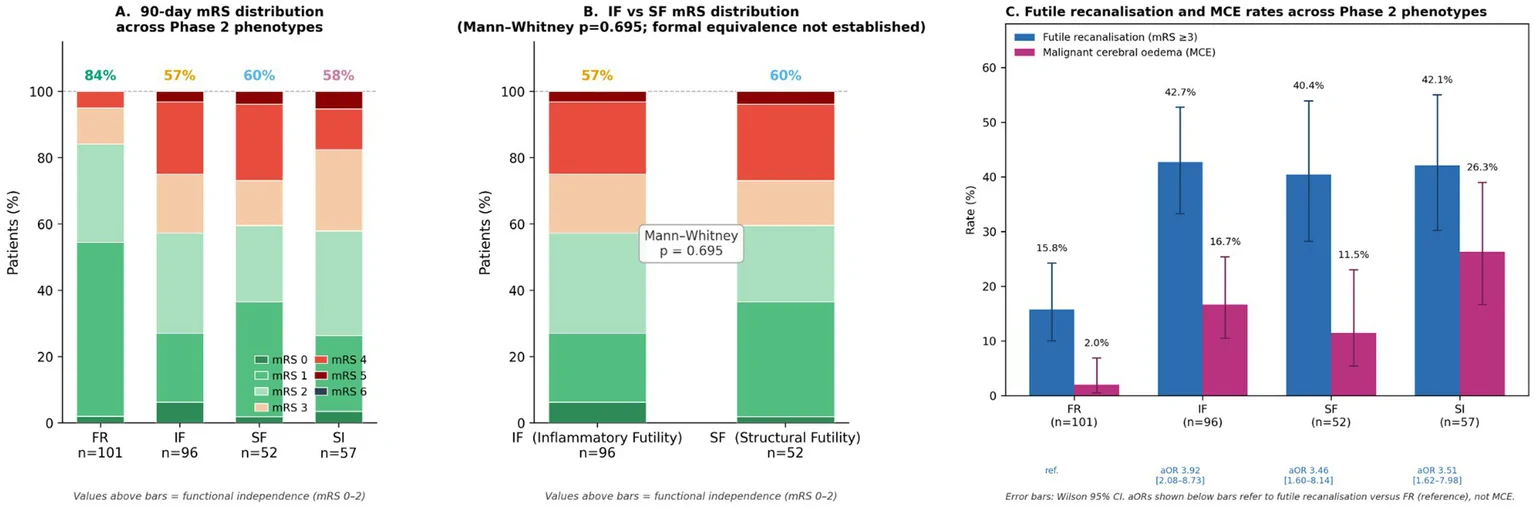
Phase 2 72-h phenotype outcomes (secondary/exploratory; *N* = 306). **(A)** 90-day mRS distribution across the four phenotypes; functional independence (mRS 0–2) annotated above each bar. **(B)** Ordinal mRS comparison between inflammatory futility and structural futility (Mann–Whitney *p* = 0.695; formal equivalence not established). **(C)** Futile recanalisation and MCE rates across phenotypes; adjusted odds ratios relative to favourable recovery are annotated. NLR₇₂ₕ threshold is dataset-derived; all Phase 2 results are exploratory. FR, favourable recovery; IF, inflammatory futility; MCE, malignant cerebral oedema; mRS, modified Rankin Scale; SF, structural futility; SI, structural-plus-inflammatory.

Futile recanalisation occurred in 102 patients (33.3%). Detailed Phase 2 phenotype results are provided in Supplementary Table S1. Functional independence (mRS 0–2) was achieved by 84.2% of favourable recovery patients (*n* = 101), 57.3% of inflammatory futility patients (*n* = 96), 59.6% of structural futility patients (*n* = 52), and 57.9% of structural-plus-inflammatory patients (*n* = 57) (Figure 4A). Adjusted odds of futile recanalisation relative to favourable recovery were 3.92 [2.08–8.73] for inflammatory futility, 3.46 [1.60–8.14] for structural futility, and 3.51 [1.62–7.98] for structural-plus-inflammatory (Figure 4C). The ordinal mRS distribution did not differ between inflammatory futility and structural futility (Mann–Whitney *p* = 0.695; formal equivalence not established) (Figure 4B).

The binary anatomy × NLR₇₂ₕ interaction was nominally significant on the Woolf test [OR 3.689 (1.336–10.188); *p* = 0.012], whereas the continuous interaction was not (*p* = 0.170), reflecting the imprecision expected in this exploratory analysis. The Harrell-corrected AUC for the Phase 2 model was 0.631 (Figure 3B). Serial NLR kinetics by Phase 2 phenotype from admission to 72 h are shown in Figure 3C. Inflammatory futility patients were more frequently assigned to the Phase 1 convergence cell than favourable recovery patients (39.6% vs. 20.8%; Fisher *p* = 0.005; Supplementary Table S3). The convergence-plus-structural-plus-inflammatory subgroup had MCE 55.0% [34.2–74.2%] (*n* = 20; hypothesis-generating).

### Prospective validation requirement

Assuming a true AUC of 0.791, MCE prevalence of 12.7%, 80% power, and two-sided *α* = 0.05, a minimum of 600 patients (approximately 76 MCE events) would be required to confirm AUC ≥ 0.70 in a prospective validation cohort. For a four-site study, this corresponds to approximately 150 patients per site, or approximately 750 patients screened after allowing for a 20% screening dropout rate.

## Discussion

Successful recanalisation after EVT does not eliminate the risk of secondary post-reperfusion injury. In this single-centre ICAD-management–predominant derivation cohort, 24-h NIHSS worsening was the dominant independent predictor of clinically ascertained MCE [aOR 6.95 (3.63–14.46); *E*-value 13.4]. However, this association is subject to incorporation bias: END was an embedded component of the clinical MCE ascertainment criteria, and adjudicators were not blinded to NIHSS trajectory; the finding should therefore not be interpreted as evidence of independent radiological prediction of cerebral oedema. NLR direction refined the complication profile of neurological deterioration rather than independently improving discrimination. The Phase 1 model achieved internally corrected discrimination of 0.791, yet the incremental AUC attributable to NLR direction beyond NIHSS worsening was minimal. NLR direction is not a standalone MCE predictor; it is a dynamic inflammatory signal that distinguishes oedema-enriched from haemorrhage-enriched post-reperfusion deterioration within the neurologically worsening subgroup. The exploratory Phase 2 analysis extended this monitoring concept to 72 h, identifying a candidate inflammatory futility phenotype in which sustained systemic inflammation despite favourable CT-perfusion anatomy was associated with unexpectedly poor functional recovery.

### Benchmarking against prior models

Most available MCE prediction models achieve only moderate discrimination using pre-procedural variables (9–11). In the present cohort, HIR alone, core volume alone, and NLR₂₄h alone each showed lower apparent discrimination than the multivariable clinical–inflammatory model (35, 36), supporting the incremental value of incorporating post-reperfusion neurological trajectory into early risk assessment. The comparison with single-predictor benchmarks must be interpreted descriptively, as single-predictor AUCs were apparent values, whereas multivariable AUCs were internally corrected. Decision curve analysis suggested superior apparent net benefit of the Phase 1 model over both the treat-all and treat-none strategies across clinically relevant threshold probabilities. These estimates are internally derived and require prospective confirmation before informing decisions on monitoring or escalation. They nonetheless reinforce the case for a bedside monitoring approach based exclusively on standard neurological assessment and routine haematological indices, adding no imaging burden or laboratory cost beyond what is routinely obtained in any post-EVT stroke unit.

### NLR direction as a complication-profile stratifier

Before discussing the biological rationale, two caveats require explicit statement. First, NLR direction did not independently improve model discrimination [ΔAUC +0.008 (95% CI −0.007 to +0.020)] and was not independently associated with MCE in the multivariable model (aOR 1.77; *p* = 0.105). Second, the complication-profile stratification described in this section is hypothesis-generating and should not be considered clinically actionable without external validation.

The choice of a directional binary operationalisation for NLR is as follows. The binary direction (ΔNLR > 0 vs. ≤0) was pre-specified because it preserves the four-cell classification structure and the Cell C vs. Cell D complication-profile distinction, the principal novel contribution of the directional framework. Supplementary Table S5 reports pre-specified alternative operationalisations: continuous ΔNLR (corrected AUC 0.796), NLR ratio (0.793), and binary direction (0.791) yielded virtually identical discrimination, supporting the robustness of the directional approach. Absolute NLR₂₄h yielded AUC 0.805, but was not pre-specified because it cannot reproduce the four-cell classification. No validated NLR direction threshold exists in the EVT literature; the ΔNLR = 0 cut-point was chosen for clinical interpretability and requires validation in external cohorts.

The biological basis of the directional NLR signal lies in the dynamics of post-reperfusion inflammation. Neutrophil trafficking after successful recanalisation contributes to blood–brain barrier disruption, matrix metalloproteinase activation, oxidative injury, and vasogenic oedema propagation (13–15). The divergent complication profiles of Cell C and Cell D show that NLR direction encodes clinically relevant information that the absolute NLR value at any single time point cannot provide.

Cell C, characterised by NIHSS worsening without NLR rise, had the highest admission NLR and a haemorrhage-enriched complication profile, relative to Cell D. This pattern is consistent with pre-existing systemic inflammatory activation predisposing to haemorrhagic vulnerability rather than acute post-reperfusion neutrophil recruitment (17, 18, 21). Conversely, Cell D, defined by concurrent NIHSS worsening and NLR rise from a low admission baseline, showed an oedema-enriched pattern consistent with post-reperfusion neutrophil trafficking and ischaemia-reperfusion-driven vasogenic oedema amplification (13–15, 21). The clinically relevant signal is therefore the inflammatory trajectory in relation to neurological deterioration, rather than the absolute NLR magnitude at any single measurement.

Patients in Cell D had nearly equal MCE and sICH rates (26.9% vs. 28.0%), whereas patients in Cell C showed sICH predominance (37.7% vs. MCE 18.9%). Although the complication-profile comparison did not reach statistical significance in this event-limited stratum, the direction of effect was biologically coherent and illustrates why AUC-based comparisons alone may underestimate the clinical relevance of NLR direction: AUC quantifies discrimination for a single endpoint and cannot characterise whether neurologically worsening patients are on an oedema or haemorrhage trajectory.

### Phase 2 and the inflammatory-futility hypothesis

The term ‘Inflammatory Futility’ requires explicit definition. As applied in this study, it designates a phenotypic pattern: technically successful recanalisation with anatomically favourable CT-perfusion parameters (core ≤30 mL and HIR ≤ 0.4, parameters ordinarily associated with expected functional recovery) (28) yet persistent systemic inflammation at 72 h (NLR₇₂ₕ above the cohort median), associated with unexpectedly poor functional outcome (90-day mRS ≥ 3). It is distinguished from structural futility—futile recanalisation attributable to unfavourable anatomy—by the presence of imaging parameters that would typically predict a favourable course. The biological hypothesis is that sustained post-reperfusion systemic inflammation, reflected by a persistently elevated NLR at 72 h, perpetuates blood–brain barrier disruption, oedema amplification, and microvascular dysfunction beyond what structural imaging predicts, thereby offsetting the survival advantage conferred by a small ischaemic core and preserved penumbral tissue. The inflammatory futility phenotype is a candidate construct requiring independent prospective validation before clinical adoption.

The most clinically pertinent observation was the inflammatory futility phenotype, in which patients with favourable anatomy but persistently elevated NLR₇₂ₕ had substantially lower functional independence than anatomically favourable patients with low NLR₇₂ₕ (57.3% vs. 84.2%). A 27-percentage-point gap in functional independence between anatomically equivalent patients stratified solely by NLR₇₂ₕ (84.2% vs. 57.3%) indicates that favourable CT-perfusion anatomy does not guarantee recovery when systemic inflammation persists. Prior studies have linked inflammatory biomarkers, including interleukin-6 and NLR, to futile recanalisation and poor functional outcome after EVT (7, 8, 37). The present cohort adds a temporal dimension by showing that inflammation at 72 h identifies patients whose outcomes are worse than CT-perfusion imaging predicts. Phase 2 findings must be interpreted strictly as exploratory: the NLR₇₂ₕ threshold was dataset-derived, the corrected AUC was modest (0.631; upper bound by construction), and the binary interaction estimate was imprecise with wide confidence intervals.

### Generalisability and ICAD-management context

The cohort was ICAD-management–predominant, operationally defined by use of rescue angioplasty, intracranial stenting, or intra-procedural tirofiban in more than half of patients. ICAD was not separately adjudicated as a standalone stroke mechanism; the findings are therefore most directly applicable to centres with comparable EVT practice patterns and high ICAD-management burden. This procedural context modulates post-reperfusion inflammatory kinetics: rescue angioplasty, stenting, plaque manipulation, endothelial injury, and antithrombotic exposure interact with the systemic ischaemia–reperfusion response in ways that amplify post-procedural neutrophil mobilisation beyond what is observed in cardioembolic stroke (19–21). In cardioembolic-predominant cohorts, the procedural inflammatory stimulus is absent, and NLR trajectories may differ accordingly. AF-related effect modification could not be reliably estimated because of sparse interaction cells at EPV = 6.50; aetiology-specific conclusions should be reserved for adequately powered prospective studies.

Of the two Phase 1 components, NIHSS worsening is expected to remain generalisable across aetiologies: neurological deterioration following large hemispheric infarction reflects common pathophysiological pathways—progressive cytotoxic and vasogenic oedema, raised intracranial pressure, and cerebral herniation—that are not mechanistically dependent on the underlying stroke mechanism or EVT technique. The NLR directional component is more context-dependent: in ICAD-management–predominant centres, the arterial inflammatory stimuli from plaque manipulation, stenting, and antithrombotic exposure may amplify post-procedural neutrophil mobilisation beyond what occurs in cardioembolic stroke, where this procedural stimulus is absent. External validation should therefore report the NIHSS-worsening and NLR-direction contributions separately in ICAD-predominant and cardioembolic-predominant cohorts to characterise the generalisability of each component independently.

### Strengths

Several methodological strengths merit comment. Temporal precedence was built into the study design: Phase 1 predictors are assigned 36–60 h before radiological MCE ascertainment becomes possible, ensuring that predictors cannot be influenced by the outcome. Low EPV was mitigated through ridge L2 penalisation and bootstrap optimism correction. Reporting follows TRIPOD standards and includes calibration metrics, decision curve estimates, reclassification indices, and the complete prediction equation with standardisation parameters to facilitate external validation (23, 30, 32). The analysis was designed from the outset to evaluate NLR direction as a complication-profile stratifier rather than as an independent discriminator, a deliberate distinction that addresses the inherent limitations of AUC-based comparisons in this context.

### Future directions

The essential next step is prospective multicentre validation with blinded, criteria-driven radiological adjudication of MCE, independent of neurological trajectory. Future studies should test whether the 24-h clinical–inflammatory framework predicts radiological oedema irrespective of NIHSS trajectory and whether the Cell C vs. Cell D complication-profile distinction is reproducible across centres with varying aetiology distributions. Validation protocols should pre-specify calibration, corrected discrimination, and clinical utility as co-primary targets, with NRI as a secondary reclassification endpoint. The Phase 2 inflammatory futility phenotype should be recalibrated in an independent cohort with separate assessment in ICAD-predominant and cardioembolic-predominant populations. Imaging-confirmed MCE without concurrent END constitutes the most important validation subgroup for clarifying whether the monitoring framework can identify oedema risk before overt neurological deterioration: a question that can only be answered by a prospective study design with protocol-driven, blinded radiological adjudication.

### Limitations

The principal limitation is the complete overlap between clinically ascertained MCE and END. All 39 MCE events co-occurred with early neurological deterioration, defined as NIHSS increase ≥4 by 72 h, reflecting the clinical component embedded in the MCE definition, including requirement for decompressive hemicraniectomy or oedema-attributable death (1, 38–40). This overlap constitutes a form of incorporation bias: because END was embedded within the MCE ascertainment criteria and adjudicators were not blinded to NIHSS trajectory, the observed association between NIHSS worsening and MCE may partly reflect how MCE was detected rather than an independent causal or radiological relationship. This is the principal threat to the internal validity of the Phase 1 framework and distinguishes it fundamentally from a model trained on radiologically confirmed MCE with independent adjudication. The Phase 1 model accordingly identifies a clinical–inflammatory risk pattern associated with MCE ascertainment, not a validated radiological oedema predictor. Prospective studies with blinded, criteria-driven radiological adjudication of MCE, independent of neurological trajectory, are required to quantify and correct for this bias.

Several statistical limitations warrant caution. The Phase 1 model included 39 events and 6 candidate predictors (EPV = 6.50); ridge-penalised effect estimates—particularly the NLR-rising estimate—should be interpreted with emphasis on direction and biological plausibility rather than precise effect magnitude. Low EPV renders model coefficients susceptible to instability due to changes in derivation sample composition. Although ridge L2 penalisation (*λ* = 1.0) was applied to stabilise estimates and Harrell bootstrap correction was used to quantify and adjust for optimism, both procedures remain internal to the derivation dataset; coefficients should be treated as preliminary estimates requiring external recalibration before clinical application. The incremental discrimination from NLR direction beyond NIHSS worsening was minimal [ΔAUC M6 − M4 = +0.008 (95% CI −0.007 to +0.020)], confirming that NLR direction does not independently improve MCE discrimination. Reclassification estimates, including NRI, should likewise be interpreted conservatively in this event-limited derivation cohort. The exploratory Cell D core-volume refinement did not retain significance after multiplicity adjustment (Bonferroni-adjusted *p* ≈ 0.13) and should be treated as hypothesis-generating.

The Phase 2 analysis was exploratory throughout. The NLR₇₂ₕ threshold was derived from the derivation cohort median, the anatomy×NLR₇₂ₕ interaction was directionally supportive but statistically imprecise, and the corrected AUC of 0.631 represents an upper bound by construction, requiring external recalibration (8, 33). The single-centre ICAD-management–predominant design limits generalisability, particularly to cardioembolic-predominant Western cohorts, and individual-level characteristics of excluded patients were unavailable for comparison. NLR is a non-specific inflammatory marker and may be confounded by infection, physiological stress, medications, procedural complexity, antiplatelet exposure, or other unmeasured inflammatory conditions. Patients with active infection at admission were excluded (*n* = 11), but hospital-acquired infections during the post-procedural monitoring period—including aspiration pneumonia and ventilator-associated pneumonia, which are common complications of severe hemispheric stroke—were not systematically screened. Because MCE itself predisposes to pulmonary infection, bidirectional confounding between NLR elevation and post-procedural infectious complications cannot be excluded; prospective validation protocols should incorporate systematic infection screening at T₁ and T₂. AF-related effect modification could not be reliably estimated because of sparse interaction cells, so neither the presence nor absence of AF as an effect modifier can be concluded. Finally, decision-curve net benefit values were derived from apparent model predictions and were not optimism-corrected; they require prospective external confirmation before informing monitoring or escalation protocols (30).

## Conclusion

In 306 consecutive patients with successful EVT, a sequential 24- and 72-h clinical-inflammatory monitoring framework enabled stratification of post-reperfusion risk before radiological confirmation. At 24 h, NIHSS worsening was the dominant marker of clinically ascertained MCE risk (corrected AUC 0.791; *E*-value 13.4), whereas the direction of NLR change identified distinct oedema-enriched and haemorrhage-enriched deterioration profiles within the neurologically worsening subgroup, a stratification that AUC-based comparisons alone cannot capture. At 72 h, persistently elevated NLR despite favourable CT-perfusion anatomy identified an exploratory inflammatory futility phenotype associated with worse-than-predicted functional recovery. The Phase 2 inflammatory futility phenotype is a candidate construct only; the NLR₇₂ₕ threshold is dataset-derived, the corrected AUC represents an upper bound by construction, and the phenotype should not be adopted clinically without external recalibration and prospective validation. Because MCE ascertainment was clinically triggered, all events co-occurred with early neurological deterioration, and adjudication was unblinded to NIHSS trajectory—constituting incorporation bias—the Phase 1 framework identifies a clinical–inflammatory risk pattern rather than a validated independent radiological predictor of cerebral oedema. Prospective multicentre validation with blinded radiological adjudication remains the required next step, and this study provides the prediction equation, standardisation parameters, and a pre-specified validation protocol needed for such evaluation.

## Data Availability

De-identified patient-level data are available upon reasonable request to the corresponding author (xjian1216@csu.edu.cn), subject to the following restrictions: (1) written approval from the Xiangya Hospital Research Governance Office; (2) a signed data-use agreement specifying the permitted research purpose and prohibiting onward redistribution; (3) removal of all direct patient identifiers before data transfer; (4) compliance with China’s Personal Information Protection Law (PIPL, 2021); and (5) independent ethics review at the recipient institution. The Python 3.12 analysis code, including all model specifications, standardisation parameters, and random seed, is available under a CC-BY 4.0 licence upon request, to permit full replication of all reported analyses.
