# Supplementary material for: Sequential NIHSS–NLR monitoring after endovascular thrombectomy: malignant cerebral oedema and inflammatory futility in a two-phase derivation study
Source: Front Neurol. 2026 Sep 3;17:1898491. doi: 10.3389/fneur.2026.1898491 (PMC13581491; doi:10.3389/fneur.2026.1898491)
Supplement: Supplementary file 1 [file Table_1.DOCX]

**Supplementary Table 1.** Phase 2 (72-Hour) Phenotype Distribution and Functional Outcomes — Secondary/Exploratory Analysis (N=306)

Phenotypes defined by CT-perfusion anatomy (core ≤30 mL AND HIR ≤0.4 = favourable) × NLR₇₂ₕ (>5.47 cohort median = high). NLR₇₂ₕ threshold is dataset-derived; all Phase 2 findings require external validation.

| **Phenotype** | **N (%)** | **Futile recanalisation n (%)** | **Functional independence n (%)** | **Median mRS [IQR]** | **MCE n (%)** | **sICH n (%)** | **OR for futile (95% CI)** | **p vs FR** |
| --- | --- | --- | --- | --- | --- | --- | --- | --- |
| Favourable Recovery (good anatomy + low NLR₇₂ₕ) | 101 (33.0%) | 22 (21.8%) | 79 (78.2%) | 2 [1–2] | 2 (2.0%) | 11 (10.9%) | 1.00 (reference) | — (reference) |
| Inflammatory Futility (good anatomy + HIGH NLR₇₂ₕ) ★ | 96 (31.4%) | 49 (51.0%) | 47 (49.0%) | 3 [2–4] | 16 (16.7%) | 17 (17.7%) | 3.40 [1.86–7.03] | <0.001 |
| Structural Futility (poor anatomy + low NLR₇₂ₕ) | 52 (17.0%) | 29 (55.8%) | 23 (44.2%) | 3 [1–4] | 6 (11.5%) | 5 (9.6%) | 3.99 [1.91–9.45] | <0.001 |
| Structural-plus-Inflammatory (poor anatomy + high NLR₇₂ₕ) | 57 (18.6%) | 36 (63.2%) | 21 (36.8%) | 3 [2–4] | 15 (26.3%) | 14 (24.6%) | 4.60 [2.21–10.43] | <0.001 |

★ Inflammatory Futility vs Structural Futility: Mann–Whitney U p=0.695 (both median mRS=3); formal equivalence not established.

Anatomy × NLR₇₂ₕ interaction: OR=2.868 [1.044–7.878]; Woolf p=0.040; continuous interaction p=0.170 (underpowered; hypothesis-generating only).

ORs adjusted for age, baseline NIHSS, admission glucose, and atrial fibrillation (bootstrap 95% CI, 2,000 iterations; seed 20250510).

† Phase 2 corrected AUC=0.631 is an upper bound by construction (threshold derived from cohort median; applying to external data without recalibration will yield optimistically biased discrimination).

Abbreviations: FR, Favourable Recovery; HIR, hypoperfusion intensity ratio; MCE, malignant cerebral œdema; mRS, modified Rankin Scale; NLR, neutrophil-to-lymphocyte ratio; sICH, symptomatic intracranial haemorrhage.
