# Supplementary material for: Sequential NIHSS–NLR monitoring after endovascular thrombectomy: malignant cerebral oedema and inflammatory futility in a two-phase derivation study
Source: Front Neurol. 2026 Sep 3;17:1898491. doi: 10.3389/fneur.2026.1898491 (PMC13581491; doi:10.3389/fneur.2026.1898491)
Supplement: Supplementary file 2 [file Table_2.DOCX]

**Supplementary Table 2.** Multivariable Logistic Regression — Phase 1 Primary Model and Phase 2 Secondary/Exploratory Model, with Decision Curve Analysis and AF Interaction Assessment

Single-centre derivation study (Xiangya Hospital, N=306); external validation required before clinical application.

| **Predictor** | **OR (95% CI)** | **p†** | **Notes** |
| --- | --- | --- | --- |
| **PHASE 1 — PRIMARY: MCE Prediction (N=306; 39 events; EPV=6.50; ridge L2 λ=1.0; Harrell-corrected; seed=20250510)** | | | |
| Intercept (log-odds) [TRIPOD 15a] | −3.6819 | — | For individual-level probability calculation, standardisation parameters below |
| Core volume, mL (per SD; μ=22.63, σ=36.10) | 1.55 [1.15–2.19] | 0.011 | β=0.4369; use z(x)=(x−μ)/σ |
| Baseline NIHSS (per SD; μ=13.06, σ=6.57) | 0.93 [0.57–1.35] | 0.735 | β=−0.0747 |
| Age, years (per SD; μ=64.18, σ=10.61) | 0.97 [0.68–1.42] | 0.875 | β=−0.0297 |
| Onset-to-groin, min (per SD; μ=537.17, σ=505.07) | 0.76 [0.41–1.09] | 0.268 | β=−0.2743 |
| NLR-rising at 24h (ΔNLR >0) | 1.77 [0.93–3.78] | 0.105 | Ridge-attenuated; directional only; β=0.5722 |
| NIHSS worsening at 24h (ΔNIHSS >0) | 6.95 [3.63–14.46] | <0.001 | Strongest predictor; E-value=13.4 (lb=6.7); β=1.9383 |
| AUC summary: M0 corrected=0.598 \| M4 corrected=0.783 \| M6 corrected=0.791 \| ΔAUC M6−M4=+0.008 [95% CI −0.007 to +0.020; CI crosses zero] — NLR-rising does NOT independently improve discrimination beyond NIHSS-worsening | | | |
| Prediction equation [TRIPOD 15b]: logit (MCE) = −3.6819 + 0.4369×z(core) + (−0.0747) ×z(NIHSS) + (−0.0297) ×z(age) + (−0.2743) ×z(OTG) + 0.5722×NLR-rising + 1.9383×NIHSS-worsening [z(x)=(x−μ)/σ] \| HL p=0.731; Brier=0.093 | | | |
| **DECISION CURVE ANALYSIS — Net Benefit (apparent values; external validation required)** | | | |
| **Risk Threshold** | **M6 Net Benefit** | **Treat-All NB** | **ΔNB (M6 − Treat-All)** |
| 5% | 0.102 | 0.082 | ΔNB=+0.021 |
| 10% | 0.077 | 0.031 | ΔNB=+0.047 |
| 15% | 0.046 | −0.027 | ΔNB=+0.073 |
| 20% | 0.027 | −0.091 | ΔNB=+0.118 |
| 25% | 0.026 | −0.163 | ΔNB=+0.190 |
| 30% | 0.022 | −0.247 | ΔNB=+0.269 |
| AF INTERACTION — INDETERMINATE: Sparse cells (7 MCE among 69 AF patients; EPV=6.50) → near-separation. Cannot conclude null effect modification. AF: MCE 10.1% vs non-AF: 13.5% (Fisher p=0.543). Aetiology-stratified external validation required. | | | |
| **PHASE 2 — SECONDARY/EXPLORATORY: Futile Recanalisation (N=306; corrected AUC=0.631 [upper bound by construction — cohort median split])** | | | |
| **Phenotype vs Favourable Recovery** | **OR (95% CI)** | **p†** | **Notes** |
| Inflammatory Futility (good anatomy + high NLR₇₂ₕ) | 3.92 [2.08–8.73] | <0.001 | Threshold-dependent; requires external recalibration |
| Structural Futility (poor anatomy + low NLR₇₂ₕ) | 3.46 [1.60–8.14] | 0.001 |  |
| Structural-plus-Inflammatory (poor anatomy + high NLR₇₂ₕ) | 3.51 [1.62–7.98] | <0.001 | Combined structural + inflammatory futility |
| Age (per SD) | 0.96 [0.74–1.25] | 0.768 | Adjusted covariate |
| Baseline NIHSS (per SD) | 1.15 [0.89–1.49] | 0.275 |  |
| Admission glucose (per SD) | 1.04 [0.81–1.33] | 0.753 |  |
| Atrial fibrillation | 1.08 [0.58–2.00] | 0.815 |  |

† p-values from Wald test (bootstrap SE). EPV=6.50; ridge L2 λ=1.0; all ORs attenuated toward null. AUC difference CI: 5,000-iteration paired bootstrap (seed 20250510).

‡ All N=306 patients had complete AF status after source-record review (AF: 69/306, 22.5%; absent: 237/306, 77.5%).

Abbreviations: AUC, Harrell-corrected; CI, 95% bootstrap; DCA, decision curve analysis; EPV, events per variable; NB, net benefit; NLR, neutrophil-to-lymphocyte ratio; TRIPOD, Transparent Reporting of a Multivariable Prediction Model for Individual Prognosis or Diagnosis.
