# Supplementary material for: Sequential NIHSS–NLR monitoring after endovascular thrombectomy: malignant cerebral oedema and inflammatory futility in a two-phase derivation study
Source: Front Neurol. 2026 Sep 3;17:1898491. doi: 10.3389/fneur.2026.1898491 (PMC13581491; doi:10.3389/fneur.2026.1898491)
Supplement: Supplementary file 3 [file Table_3.DOCX]

**Supplementary Table 3.** Cross-Tabulation of Phase 1 Cell Assignment and Phase 2 Phenotype: Patient Distribution and MCE Rates (N=306)

MCE rates shown as n/% [Wilson 95% CI] within each joint cell. Post-hoc exploratory analysis requires prospective confirmation.

| **Phase 1 Cell (24-hour)** | **Favourable Recovery (n=101)** | **Inflammatory Futility (n=96)** | **Structural Futility (n=52)** | **Structural-plus-Inflammatory (n=57)** | **Row Total (Phase 1 MCE)** |
| --- | --- | --- | --- | --- | --- |
| Reference cell (NLR-non-rising + NIHSS-non-worsening; n=72) | n=36, MCE=0 [0.0–9.6%] | n=13, MCE=0 [0.0–22.8%] | n=17, MCE=0 [0.0–18.4%] | n=6, MCE=0 [0.0–39.0%] | N=72, MCE=0 (0.0%) |
| Cell B (NLR-rising + NIHSS-non-worsening; n=88) | n=36, MCE=0 [0.0–9.6%] | n=26, MCE=0 [0.0–12.9%] | n=15, MCE=1/6.7% [1.2–29.8%] | n=11, MCE=3/27.3% [9.7–56.6%] | N=88, MCE=4 (4.5%) |
| Cell C (NLR-non-rising + NIHSS-worsening; n=53) | n=8, MCE=1/12.5% [2.2–47.1%] | n=19, MCE=6/31.6% [15.4–54.0%] | n=6, MCE=2/33.3% [9.7–70.0%] | n=20, MCE=1/5.0% [0.9–23.6%] | N=53, MCE=10 (18.9%) |
| Convergence cell (NLR-rising + NIHSS-worsening; n=93) | n=21, MCE=1/4.8% [0.8–22.7%] | n=38, MCE=10/26.3% [15.0–42.0%] | n=14, MCE=3/21.4% [7.6–47.6%] | n=20, MCE=11/55.0% [34.2–74.2%] | N=93, MCE=25 (26.9%) |
| Column Total (Phase 2 MCE rate) | N=101, MCE=2 (2.0%) | N=96, MCE=16 (16.7%) | N=52, MCE=6 (11.5%) | N=57, MCE=15 (26.3%) | N=306, MCE=39 (12.7%) |

Key statistics: IF patients in convergence cell 38/96 (39.6%) vs FR 21/101 (20.8%); Fisher p=0.005. Overall Phase 1 × Phase 2 distribution: χ² p<0.001.

Highlighted joint cell of clinical interest: Convergence × Inflammatory Futility (n=38); MCE rate 26.3% — highest oedema risk among anatomically favourable patients.

Abbreviations: FR, Favourable Recovery; IF, Inflammatory Futility; MCE, malignant cerebral œdema; NLR, neutrophil-to-lymphocyte ratio; SF, Structural Futility; SI, Structural-plus-Inflammatory.
