# Supplementary material for: Sequential NIHSS–NLR monitoring after endovascular thrombectomy: malignant cerebral oedema and inflammatory futility in a two-phase derivation study
Source: Front Neurol. 2026 Sep 3;17:1898491. doi: 10.3389/fneur.2026.1898491 (PMC13581491; doi:10.3389/fneur.2026.1898491)
Supplement: Supplementary file 4 [file Table_4.DOCX]

**Supplementary Table 4.** MCE Prediction Benchmark: M6 (Harrell-Corrected AUC=0.791) vs Single Peri-Procedural Predictors (Apparent AUC)

Single-predictor AUCs are apparent (no Harrell correction). Multivariable model AUCs are Harrell-corrected (seed=20250510; 1,000 resamples). Comparison is conservative for M6: equal correction applied to single predictors would widen ΔAUC further. Benchmark comparisons are descriptive only.

| **Predictor / Model** | **AUC** | **AUC type** | **ΔAUC vs M6** | **Note** |
| --- | --- | --- | --- | --- |
| HIR alone | 0.608 | Apparent | +0.183 | CT perfusion; DAWN/DEFUSE eligibility parameter |
| Core volume alone | 0.659 | Apparent | +0.132 | CT perfusion: primary structural predictor |
| Baseline NIHSS alone | 0.577 | Apparent | +0.214 | Admission clinical severity |
| NLR₂₄h alone | 0.688 | Apparent | +0.103 | Post-reperfusion NLR absolute value |
| ΔNLR (T₁−T₀) | 0.643 | Apparent | +0.148 | Absolute NLR change over 24h |
| M0: Base model | 0.598 | Harrell-corrected | +0.193 | 4 predictors; no post-reperfusion data |
| M4: M0 + NIHSS-worsening | 0.783 | Harrell-corrected | +0.008 | 5 predictors; NIHSS trajectory only |
| M6: Primary model | 0.791 | Harrell-corrected | — | 6 predictors; EPV=6.50; ridge L2 λ=1.0; canonical |

Note on ΔAUC M6−M4: Paired bootstrap ΔAUC=+0.008 [95% CI −0.007 to +0.020] (5,000 iterations; seed 20250510); CI crosses zero. NLR-rising does not independently improve discrimination beyond NIHSS-worsening.

NRI of M6 vs HIR alone: categorical 0.658 [0.382–0.899]; continuous 0.863 [0.563–1.131] (Supplementary Table S7).

Abbreviations: AUC, area under ROC curve; HIR, hypoperfusion intensity ratio; NLR, neutrophil-to-lymphocyte ratio.
