# Supplementary material for: Sequential NIHSS–NLR monitoring after endovascular thrombectomy: malignant cerebral oedema and inflammatory futility in a two-phase derivation study
Source: Front Neurol. 2026 Sep 3;17:1898491. doi: 10.3389/fneur.2026.1898491 (PMC13581491; doi:10.3389/fneur.2026.1898491)
Supplement: Supplementary file 5 [file Table_5.DOCX]

**Supplementary Table 5.** Binary vs Continuous NLR Operationalisation in M6 (Harrell-Corrected)

All AUCs are Harrell-corrected using a 1,000-resample bootstrap (seed=20250510). Differences are relative to the pre-specified binary direction operationalisation.

| **NLR operationalisation in M6** | **Corrected AUC** | **Δ vs binary** | **Apparent AUC** | **Conclusion** |
| --- | --- | --- | --- | --- |
| NLR-rising (binary; ΔNLR>0) — pre-specified | 0.791 | — | 0.791 | Reference: preserves four-cell complication-profile classification matrix |
| Continuous ΔNLR (T₁−T₀) | 0.796 | +0.005 | 0.796 | Non-inferior; binary loses <0.01 AUC |
| NLR ratio (T₁/T₀) | 0.793 | +0.002 | 0.793 | Non-inferior; relative-change metric |
| Continuous NLR₂₄h (absolute value) | 0.805 | +0.014 | 0.805 | Cannot generate four-cell matrix or Cell C vs D stratification; not pre-specified |

ΔNLR in MCE patients: median +3.84 vs No-MCE: +0.47 (p=0.004). NLR ratio: MCE 2.03 vs No-MCE 1.13 (p=0.003). Continuous NLR₂₄h gains +0.014 AUC but cannot reproduce the four-cell complication-profile classification and was not pre-specified.

Abbreviations: AUC, Harrell-corrected area under the ROC curve; NLR, neutrophil-to-lymphocyte ratio.
