# Supplementary material for: Sequential NIHSS–NLR monitoring after endovascular thrombectomy: malignant cerebral oedema and inflammatory futility in a two-phase derivation study
Source: Front Neurol. 2026 Sep 3;17:1898491. doi: 10.3389/fneur.2026.1898491 (PMC13581491; doi:10.3389/fneur.2026.1898491)
Supplement: Supplementary file 6 [file Table_6.DOCX]

**Supplementary Table S6. Exploratory Sub-Analyses: Cell D Core-Volume Refinement, IVT Effect, First-Pass Success, and LVO Location**

All analyses are post-hoc and exploratory; no multiple comparison correction was applied. Findings should be treated as hypothesis-generating and require prospective validation. The 10 mL core-volume threshold for Cell D refinement was selected post-hoc and requires recalibration in external cohorts.

**Section A: Cell D (Convergence) Refinement by Ischaemic Core Volume (Exploratory; threshold post-hoc; requires external validation)**

| **Subgroup** | **n** | **MCE n (%)** | **Wilson 95% CI** | **Note** |
| --- | --- | --- | --- | --- |
| Cell D — all (reference) | 93 | 25 (26.9%) | [18.9–36.7%] | Convergence phenotype overall (MCE 26.9%) |
| **Cell D + core volume ≥10 mL** | **41** | **16 (39.0%)** | **[25.7–54.3%]** | **Extreme-risk subgroup (Fisher p=0.033 vs <10 mL)** |
| Cell D + core volume <10 mL | 52 | 9 (17.3%) | [9.4–29.7%] | Lower-risk subgroup within convergence cell |

Fisher p (core ≥10 mL vs <10 mL within Cell D) = 0.033. Core volume ≥10 mL threshold was selected post-hoc after examining thresholds of 5, 10, 15, and 20 mL; the 10 mL threshold gave the strongest Fisher p (p=0.033). This threshold coincides with DAWN trial eligibility criteria (core ≤10 mL = "good"). Correction for multiple threshold comparisons would increase the effective p-value.

**Section B: IV Thrombolysis (IVT) and MCE Risk (Exploratory; no statistically significant interaction detected)**

| **Group** | **n** | **MCE n (%)** | **Wilson 95% CI** | **Note** |
| --- | --- | --- | --- | --- |
| IVT recipients | 75 | 13 (17.3%) | [10.4–27.4%] | Fisher p vs no-IVT: 0.169; not statistically significant |
| No IVT (EVT alone) | 231 | 26 (11.3%) | [7.8–16.0%] | Reference: lower MCE rate observed |

**Section C: First-Pass Success and MCE Risk (Exploratory; trend in expected direction; p not significant)**

| **Group** | **n** | **MCE n (%)** | **Wilson 95% CI** | **Note** |
| --- | --- | --- | --- | --- |
| First-pass success (eTICI ≥2b in ≤1 pass) | 43 | 3 (7.0%) | [2.4–18.6%] | Fisher p vs multi-pass: 0.323; trend in expected direction |
| Multi-pass (>1 pass required) | 263 | 36 (13.7%) | [10.1–18.4%] | Procedural complexity may amplify the inflammatory signal |

**Section D: LVO Location and MCE Risk (Exploratory; ICA/M1 vs M2/other)**

| **LVO Location** | **n** | **MCE n (%)** | **Wilson 95% CI** | **Note** |
| --- | --- | --- | --- | --- |
| ICA terminus / M1 segment | 247 | 34 (13.8%) | [10.0–18.6%] | Fisher p vs M2/distal: 0.384; larger territory at risk |
| M2 / distal segment | 59 | 5 (8.5%) | [3.7–18.4%] | Smaller territory; lower absolute oedema risk |
