# Supplementary material for: Sequential NIHSS–NLR monitoring after endovascular thrombectomy: malignant cerebral oedema and inflammatory futility in a two-phase derivation study
Source: Front Neurol. 2026 Sep 3;17:1898491. doi: 10.3389/fneur.2026.1898491 (PMC13581491; doi:10.3389/fneur.2026.1898491)
Supplement: Supplementary file 7 [file Table_7.DOCX]

**Supplementary Table S7. Net Reclassification Improvement (NRI): M6 vs Single Predictors and M4**

Categorical NRI uses cut-points 10%, 20%, and 30% predicted probability. Continuous NRI (Pencina et al.) uses all probability differences. Bootstrap 95% CIs: 2,000 iterations; seed 20250510. Canonical M6 AUC=0.791.

| **Comparison** | **Categorical NRI** | **95% CI** | **Continuous NRI** | **95% CI** | **Interpretation** |
| --- | --- | --- | --- | --- | --- |
| **M6 vs HIR alone** | **0.658** | **[0.382–0.899]** | **0.863** | **[0.563–1.131]** | **56.4% of MCE patients were correctly reclassified upward** |
| M6 vs Core volume alone | 0.470 | [0.228–0.682] | 1.002 | [0.735–1.223] | Large reclassification benefit over structural imaging alone |
| M6 vs M4 (NIHSS-worsening alone) | 0.173 | [0.007–0.343] | 0.349 | [0.031–0.639] | NLR adds reclassification beyond NIHSS; CI excludes zero |

NRI>0 indicates net improvement in classification relative to the reference model. Categorical NRI at 10/20/30% cut-points represents clinically relevant decision thresholds. All analyses are post-hoc and exploratory.

Abbreviations: CI, 95% bootstrap confidence interval; HIR, hypoperfusion intensity ratio; NRI, net reclassification improvement; NLR, neutrophil-to-lymphocyte ratio.
